# Side Effects of Opioids Are Ameliorated by Regulating TRPV1 Receptors

**DOI:** 10.3390/ijerph19042387

**Published:** 2022-02-18

**Authors:** Xiaqing Wang, Chongyu Bao, Zhenjiang Li, Lupeng Yue, Li Hu

**Affiliations:** 1 CAS Key Laboratory of Mental Health, Institute of Psychology, Beijing 100101, China; wangxq@psych.ac.cn (X.W.); baocy@psych.ac.cn (C.B.); seanlisince1995@126.com (Z.L.); 2 Department of Psychology, University of Chinese Academy of Sciences, Beijing 100049, China

**Keywords:** TRPV1, μ-opioid, opioid side effects, neuropathic pain, β-arrestin2

## Abstract

Humans have used opioids to suppress moderate to severe pain for thousands of years. However, the long-term use of opioids has several adverse effects, such as opioid tolerance, opioid-induced hyperalgesia, and addiction. In addition, the low efficiency of opioids in controlling neuropathic pain limits their clinical applications. Combining nonopioid analgesics with opioids to target multiple sites along the nociceptive pathway may alleviate the side effects of opioids. This study reviews the feasibility of reducing opioid side effects by regulating the transient receptor potential vanilloid 1 (TRPV1) receptors and summarizes the possible underlying mechanisms. Blocking and activating TRPV1 receptors can improve the therapeutic profile of opioids in different manners. TRPV1 and μ-opioid receptors are bidirectionally regulated by β-arrestin2. Thus, drug combinations or developing dual-acting drugs simultaneously targeting μ-opioid and TRPV1 receptors may mitigate opioid tolerance and opioid-induced hyperalgesia. In addition, TRPV1 receptors, especially expressed in the dorsal striatum and nucleus accumbens, participate in mediating opioid reward, and its regulation can reduce the risk of opioid-induced addiction. Finally, co-administration of TRPV1 antagonists and opioids in the primary action sites of the periphery can significantly relieve neuropathic pain. In general, the regulation of TRPV1 may potentially ameliorate the side effects of opioids and enhance their analgesic efficacy in neuropathic pain.

## 1. Introduction

Humans have used opioids for thousands of years to suppress pain or gain euphoria. At present, opioids are still the mainstay of acute pain management, but their long-term use has severe side effects, including opioid tolerance, opioid-induced hyperalgesia, dependence, and addiction [1]. Nevertheless, opioids remain one of the most widely prescribed medications for patients with chronic pain in the USA, contributing to the opioid crisis [2]. Moreover, the analgesic efficacy of opioids is significantly lower in neuropathic pain than in other painful conditions [3,4].

The opioid receptor family is composed of three members, the µ, δ, and κ opioid receptors, that respond to classical opioid alkaloids [5]. The most commonly used opioids are agonists of μ-opioid receptors, which transduce two parallel and dissociable signaling cascades: G protein signaling pathways and β-arrestin-mediated signaling pathways [6]. The μ-opioid receptors are located presynaptically on central terminals of primary afferent neurons and postsynaptic dorsal horn neurons [7,8,9]. In addition, the μ-opioid receptor G protein signaling pathway contributes to the analgesic effect by coupling to Gαi and Gαo, which suppresses the production of cyclic adenosine monophosphate (cAMP) [10]. However, the β-arrestin signaling can be involved in the adverse effects of opioids, especially the tolerance to opioid antinociception [1,11]. For instance, functional deletion of the β-arrestin 2 gene in mice remarkably enhanced the analgesic effect of morphine and failed to develop antinociceptive tolerance [11,12]. As the pivotal negative regulators and scaffolds of GPCR signaling, β-arrestin signaling phosphorylates the μ-opioid receptor via G protein-coupled receptor kinases (GRKs) and inhibits G protein signaling-mediated analgesia [1,6].

Reducing the side effects of opioids is crucial to improving pain management. The transmission of pain signals involves a multitude of parallel processes; thus, a single-target analgesic may not be as effective as expected. Combining nonopioid analgesics with opioids to target multiple sites along the nociceptive pathway may reduce the opioid dose, improve the analgesic effect, and enhance safety [1,13].

Transient receptor potential vanilloid type 1 (TRPV1) is a highly validated pain target because its agonists and antagonists are potential analgesics in clinical trials [14,15]. TRPV1, a transient receptor potential ion channel family member, is a molecular detector for physical stimuli. It can be activated by noxious heat (≥43 °C), some endogenous lipid-derived molecules, acidic solutions (pH < 6.5), some pungent chemicals or food ingredients such as capsaicin, and toxins such as resiniferatoxin and vanillotoxins [16]. TRPV1 is mainly expressed on the peripheral and central terminals of small-diameter sensory neurons (nociceptors) in the dorsal root ganglion (DRG), trigeminal ganglion, geniculate ganglion, nodose ganglion, and jugular ganglion [17,18], and also expressed at the low level in the brain [19,20]. The activation of TRPV1 induces pain-related behaviors, and the importance of TRPV1 in nociception is also validated by deletion of the TRPV1 gene (knock-out mice) and knock-down of TRPV1 by RNA interference [21]. Previous studies also showed that antagonism of TRPV1 relieves thermal and/or mechanical hypersensitivity in multiple models of inflammation induced by ultraviolet B, complete Freund’s adjuvant, and postoperative or bone cancer pain in rodent models and humans [22,23]. Similar results were also found in neuropathic pain conditions. For instance, blockade of TRPV1 with AS1928370 [24] or capsazepine [25] ameliorates the neuropathic pain evoked by nerve ligation in rats.

Previous reports showed that TRPV1 receptors often co-expressed with μ-opioid receptors in peripheral nervous tissues, including dorsal root ganglion cells, and also expressed at the low level in the brain [26,27,28]. A reciprocal interaction possibly exists between opioid and TRPV1 receptors. For instance, chronic morphine exposure increases TRPV1 expression in the spinal cord, DRG, and sciatic nerve [29,30], whereas blockade of TRPV1 with its antagonist SB366791 potentiates the analgesic effects of systemic morphine in mice with bone cancer [31]. Similarly, another TRPV1 antagonist, capsazepine, can also strengthen morphine antinociception in mice [32]. Moreover, a growing body of evidence suggests that TRPV1 is closely linked to the adverse effects of opioids, especially opioid tolerance and opioid-induced hyperalgesia [33]. Intrathecal or intraperitoneal pretreatment with the TRPV1 antagonist SB366791 or capsazepine reduces morphine tolerance and the associated thermal hyperalgesia [29]. In addition, blocking TRPV1 receptors in the reward system, particularly expressed in the striatum and nucleus accumbens (NAc), attenuates opiate-mediated reward behaviors, including conditioned place preference (CPP) and morphine self-administration in rodents [34,35].

Therefore, we summarize the role of TRPV1 in antinociception and its potential to reduce the side effects of opioids, such as opioid tolerance, opioid-induced hyperalgesia, addiction, and inefficiency in the mitigation of neuropathic pain. Our review may provide researchers with a concise and clear perspective to weigh the value and potency of TRPV1 as an auxiliary target in pain relief.

## 2. Opioid Tolerance and Opioid-Induced Hyperalgesia

Long-term opioid use leads to prominent opioid tolerance, which means less susceptibility to opioids, and requires a higher dosage to achieve the same analgesic effect. With the increase in dose, the phenomenon of a paradoxically increased sensitivity to pain rather than relief of pain may occur, that is, opioid-induced hyperalgesia [1,36]. In this case, decreasing the dosage of opioid or discontinuing it and using other strategies are needed to maintain continuous analgesia. Although the mechanisms of opioid tolerance and opioid-induced hyperalgesia remain unclear, several overlapping pathways are implicated in opioid tolerance and opioid-induced hyperalgesia [37], including TRPV1 receptors [26,29]. For example, chronic morphine treatment induces opioid tolerance and tolerance-associated thermal hyperalgesia, concomitant with the increase in TRPV1 expression in the spinal cord, DRG, and sciatic nerve of rats [29]. Repeated co-treatment of capsazepine (a TRPV1 antagonist, 2.5 mg/kg i.p.) with morphine alleviates the development of opioid tolerance evaluated by the hot-plate test in naive mice [32]. Similarly, oral administration of AMG0347, another TRPV1 antagonist, reverses thermal and tactile hypersensitivity induced by repetitive morphine administration in naive mice and rats [30]. In addition, deleting TRPV1-expressing afferent neurons by the resiniferatoxin, ultrapotent capsaicin, attenuates the development of morphine analgesic tolerance [38]. Repeated administration of morphine by subcutaneous implantation of morphine pellets evokes thermal and tactile hypersensitivity in wild-type mice but not in TRPV1 receptor knock-out mice [30]. These studies suggest that blocking TRPV1 receptors reduces opioid tolerance and opioid-induced hyperalgesia to some extent.

Interestingly, activation of TRPV1 has the potential to mitigate opioid tolerance and improve the therapeutic profile of opioids by inhibiting μ-opioid receptor phosphorylation while leaving intact the analgesic pathway mediated by G protein signaling [30]. There are 11 phosphorylation sites on the C terminus of μ-opioid receptors, such as serine, tyrosine, and threonine, interfacing with the G protein [39]. The phosphorylation of sites on the C-terminus of μ-opioid receptors causes acute desensitization and the development of long-term opioid tolerance [39]. In addition, phosphorylation of the cytoplasmic domain of GPCRs promotes the recruitment of β-arrestins to the plasma membrane and the association of β-arrestins with GPCRs [40]. During the activation of TRPV1, calcium influx through TRPV1 improves a calcium/calmodulin-dependent translocation of G protein-coupled receptor kinase 5 shuttling from the plasma membrane to the nucleus in transfected cells, thus blocking its ability to phosphorylate μ-opioid receptors [26]. A recent study revealed that TRPV1 activation stimulated a mitogen-activated protein kinase (MAPK) signaling pathway, along with the shuttling of the scaffold protein β-arrestin2 to the nucleus [41]. This phenomenon hinders the recruitment of β-arrestin2 to μ-opioid receptors and blocks the subsequent internalization, thereby minimizing μ-opioid receptors’ desensitization and prolonging morphine-induced antinociception [41]. In summary, the activation of TRPV1 with agonists facilitates the antinociception of opioids.

TRPV1 is also dynamically regulated by β-arrestin2, and associates with scaffolds phosphodiesterase PDE4D5, which decreases cyclic AMP levels, leading to the desensitization of TRPV1 via reduced protein kinase A (PKA) activity [42]. A cross-talk also occurs between GPCRs and TRPV1 in a β-arrestin2-dependent manner. Rowan et al. demonstrated that chronic activation of peripheral µ-opioid receptors with prototypical µ-opioid receptor selective agonists (DAMGO and morphine) leads to the recruitment of β-arrestin2 to µ-opioid receptors and away from TRPV1, simultaneously causing the sensitization of TRPV1 in primary sensory neurons and contributing to behavioral symptoms of opioid-induced hyperalgesia [32]. Conversely, the highly selective µ-opioid receptor agonist herkinorin, which produces full agonist responses but without β-arrestin2 recruitment, exerts no effect on TRPV1 sensitivity [43]. In brief, β-arrestin2 may play a pivotal role in the bidirectional cross-talk between μ-opioid receptors and TRPV1 receptors.

Overall, blocking and activating TRPV1 can improve the therapeutic profile of opioids in different manners. Blocking TRPV1 improves opioid-induced analgesia and reduces opioid tolerance, possibly by neutralizing TRPV1 sensitization, whereas activating TRPV1 prevents μ-opioid receptor desensitization and retains sufficient functional μ-opioid receptors. Thus, drug combinations, developing dual-acting drugs that simultaneously target μ-opioid and TRPV1 receptors, or drugs with β-arrestin2-biased TRPV1 receptors may be more effective in controlling pain.

Notably, because of the important role of TRPV1 in thermoregulation, most of the drugs targeting TRPV1 receptors have an effect on body temperature, hyperthermia, or hypothermia. The good news is that, although TRPV1 can be activated by capsaicin (CAP), protons (low pH), and heat, only one mode of TRPV1 activation—by protons—is involved in thermoregulatory responses [44]. In contrast to the first-generation (polymodal) TRPV1 antagonists, which potently block all three TRPV1 activation modes [44], the second-generation (mode-selective) TRPV1 antagonists potently block channel activation by CAP, but exert different effects in the proton and heat modes. Some of them produce hypothermia or have no effect on body temperature [44,45]. A recent report also suggested that treatment with an intrathecal TRPV1 antagonist (SB366791) had no significant effect on body temperature [46]. Thus, developing modality-specific TRPV1 antagonists or site-specific TRPV1 antagonists is highly likely to overcome those setbacks [44,47]. Moreover, due to the poor water solubility of capsaicin derivatives, increasing its bioavailability should not be omitted [45].

## 3. Addiction

Repeated exposure to opioids induces drug addiction because of their powerful rewarding properties [48,49]. Drug addiction (or substance addiction) is a neuropsychiatric disorder characterized by compulsive drug-seeking despite the adverse health, economic, and social consequences [50]. Even though the explicit molecular and cellular mechanisms underlying morphine addiction remain unclear, the long-term neural adaptations in specific brain reward regions have been revealed [51], and numerous proteins change their expression pattern following morphine treatment [52,53]. Previous reports showed that TRPV1 is also expressed in the brain [54,55], including the nucleus accumbens (NAc) [56] and dorsal striatum (DSt) [35], which are involved in drug addiction. In addition, growing evidence suggests that TRPV1 is involved in the behavioral and neuronal adaptations evoked by addictive agents, including morphine, cocaine, ethanol, and others [35,57,58].

For instance, treatment with TRPV1 agonists systemically potentiates morphine reward, whereas pretreatment with TRPV1 antagonists attenuates these effects as assessed by the conditioned place preference (CPP) test [35]. In addition, repeated morphine treatments upregulate TRPV1 expression in the DSt, and microinjection of a selective TRPV1 antagonist (SB366791) into the DSt significantly attenuates morphine-induced CPP [35]. The same result is found in the NAc; for example, TRPV1 knock-out mice do not exhibit morphine reward responses, and intraperitoneal and intra-NAc injections of SB366791 reduce morphine-induced CPP in wild-type mice [56]. Furthermore, TRPV1 has a consistent effect in the morphine self-administration paradigm [34], which may relevantly model drug-taking behavior in a natural environment and be regarded as a gold-standard indicator of addiction [59]. Blocking the TRPV1 receptor with SB366791 significantly reduces morphine self-administration in rats on a fixed-ratio 1 schedule or a progressive ratio schedule of reinforcement, i.e., suppresses the motivational properties of morphine and its reinstatement or relapse [34]. Moreover, treatment with AMG9810, another selective TRPV1 antagonist, does not merely notably inhibit morphine self-administration but also suppresses morphine-induced c-fos expression in the NAc [34]. TRPV1 antagonist-treated rats also show decreased anxiety-like behaviors after abstinence from morphine and alleviate negative emotions during withdrawal [34]. In general, TRPV1 participates in mediating opioid reward, especially in the DSt and NAc.

Recently, a few studies have explored the possible mechanisms by which TRPV1 contributes to morphine addiction [35,60]. Nguyen et al. demonstrated that repeated morphine treatment increases μ-opioid receptor binding and the expression of adenylyl cyclase 1 (AC1), p38 mitogen-activated protein kinase (p38 MAPK), and nucleus factor kappa B (NF-κB) in the DSt; by contrast, these phenomena are suppressed by the TRPV1 antagonist capsazepine. In addition, TRPV1 is involved in the mediation of p38/NF-κB signaling, which plays a crucial role in reward-related learning and memory of addictive drugs [61,62,63]. In addition, the inhibitors of p38 and NF-kB can inhibit TRPV1 activation and morphine-induced CPP [35]. Similar mechanisms are also shown in the NAc. For example, infusion of SB366791, another selective TRPV1 antagonist into the NAc, suppresses morphine-CPP behavior and induces the phosphorylation of p38 MAPK, NF-κB, and adenylyl cyclase 1 in the NAc in morphine-treated mice. All the changes are reproduced by the selective p38 MAPK inhibitor SB203580 [56]. A follow-up study demonstrated that blocking TRPV1 with SB366791 observably decreases the activation of calmodulin-dependent protein kinase II (CaMKII), Akt, and the cAMP response element-binding protein (CREB) in the NAc evoked by morphine self-administration but exerts no significant effect on the expression levels of phospho-Protein kinase A and phospho-protein kinase C [60].

The underlying neural and molecular mechanisms of TRPV1 receptors in opioid addiction are complex and diverse, and most of the studies we refer to are from the same laboratory. Thus, additional studies should be designed for thorough investigation. Collectively, blocking TRPV1 receptors expressed in the dorsal striatum and nucleus accumbens attenuates opiate-mediated reward behaviors, including CPP and morphine self-administration in rodents, which may provide new insights about the role of TRPV1 in opioid addiction [34,35]. In addition, TRPV1 is also expressed and activated in other regions, which are closely involved in reward and drug addiction, including the frontal cortex, ventral tegmental area, and hippocampus [64,65]; however, few studies have focused on investigating this issue.

## 4. In Neuropathic Pain

Neuropathic pain is caused by diseases or lesions of the somatosensory nervous system. It is classified as central or peripheral, according to the site of the lesions or diseases causing pain [66]. Various pathologies may cause neuropathic pain, such as infection, diabetes, nerve trauma or compression, and autoimmune disease [67], which increases treatment difficulty and disturbs a significant proportion of people. Unlike the powerful efficacy of opioids in relieving inflammatory pain, they are inefficient in controlling neuropathic pain [68,69,70]. A recent study even suggested that sub-chronic pain treatment with systemic morphine dramatically exacerbates cold and mechanical allodynia in nerve-injured mice [71]. Furthermore, multiple mechanisms are posited to be responsible for the reduced opioid antinociceptive efficacy; nerve injury triggers glial cell activation and promotes the production of numerous pronociceptive factors, thereby reducing the analgesic effect [72,73]. Some researchers argue that the reduced analgesic efficacy of systemic morphine is due to the lack of functional spinal µ-opioid receptors following nerve injury [74,75]. Furthermore, recent findings indicate that the degradation of µ-opioid receptors in sensory neurons in rats with diabetic neuropathy impairs the inhibitory effects of opioids on capsaicin-induced TRPV1 activity [76]. Due to the complex mechanisms of neuropathic pain and the resistance to opioid analgesics, novel therapeutic drugs or targets must be explored to relieve neuropathic pain.

Accumulating evidence suggests that TRPV1 plays a crucial role in neuropathic pain. TRPV1 expression increases at the mRNA and protein levels in rats with chronic constriction injury (CCI), especially in small-to-medium neurons [77]. Moreover, the sensitization of spinal TRPV1 is enhanced and contributes to the development or maintenance of mechanical allodynia in CCI-treated rats [78]. Silencing spinal TRPV1 with small interference RNA (siRNA) attenuates the neuropathic pain induced by CCI by inhibiting Ca^2+^/CaMKII expression and extracellular signal-regulated kinase 2 phosphorylation [79]. Some studies revealed that systemic or central injection of TRPV1 antagonists alleviates mechanical sensitivity in neuropathic pain models [23,80,81] evoked by nerve ligation.

Various strategies to relieve neuropathic pain are associated with suppressing TRPV1 upregulation; for instance, electroacupuncture [82] alleviates paclitaxel-induced peripheral neuropathic pain in rats, possibly by suppressing TRPV1 upregulation and TLR4 signaling in DRG neurons. Similarly, Luo et al. revealed that zinc prevents paclitaxel-induced mechanical hypersensitivity in male and female mice by inhibiting the TRPV1 channel, and that extracellularly applied zinc inhibits the function of TRPV1 expressed in HEK293 cells and mouse DRG neurons [83]. The antinociceptive role of oxytocin in neuropathic pain is closely related to the inhibition of TRPV1 activation in the spinal cord [84]. Interestingly, De Gregorio et al. found the analgesic effect of cannabidiol is predominantly mediated by TRPV1 activation; the antiallodynic effects of CBD are prevented by capsazepine (10 mg/kg/day, s.c., for 7 days) in rats with neuropathic pain [85]. Furthermore, the analgesic effect of activin C is abolished in TRPV1 knock-out mice with CCI [86]. In brief, regulating the TRPV1 channel has the potential to relieve neuropathic pain.

In neuropathy, the input from peripheral sensory afferents contributes to the induction of pain, maintenance of pain, and central sensitization [67,87]. This result indicates that targeting peripheral nerves may suppress the noxious drive and the accompanied central sensitization, and provide a novel analgesic strategy by circumventing the adverse effects of systemic and centrally acting drugs [88,89]. Although TRPV1 is also expressed in the central nervous system, it is primarily expressed in peripheral nociceptive neurons [90,91]. Therefore, regulation of peripheral TRPV1 is a good choice for pain intervention, such as the capsaicin 8% dermal patch, which is a currently available option for patients with peripheral neuropathic pain [92]. Furthermore, sustained suppression of TRPV1 by local injection of zinc can reduce chemotherapy-induced neuropathic pain for >4 days after a single injection [83]. In addition, Labuz et al. found that the blockade of TRPV1 at the peripheral endings (injured nerve-innervated paw) had more profound analgesia in sciatic nerve chronic constriction-injured mice compared with that at the trunk of the injured nerve [93]. This finding contradicts opioids, which are faint and even ineffective when injected into the injured nerve-innervated paw. Furthermore, co-administration of opioids and TRPV1 antagonists at both locations partially decreases neuropathy-evoked heat and mechanical pain [93].

In brief, neuropathic pain is complex and hard to alleviate. Thus, combining drugs is preferred to integrate simultaneous actions on multiple targets. According to the different primary action sites of TRPV1 antagonists and opioid receptor agonists, co-administration of opioids and TRPV1 antagonists in the key sites of the injured nerve peripheral terminals or the nerve trunk can be helpful to relieve neuropathic pain.

## 5. Discussion

The role of TRPV1 in pain modulation is well known, and a growing body of research has clarified the reciprocal interaction between opioid and TRPV1 receptors [42]. Thus, we reviewed the feasibility of compensating for the defects of opioids by regulating TRPV1 and summarized the potential underlying mechanisms, as described in Table 1.

As shown in Table 1, the crucial role of TRPV1 in these processes indicates that TRPV1 may be a candidate target to strengthen opioid analgesia and reduce the side effects. Emerging research has verified the validity of this multi-target drug strategy. Recently, Lee et al. designed a dual-acting scaffold by combining the key pharmacophores of the potent TRPV1 antagonist (GRT12360) and the piperidine MOR agonist (Meperidine) to generate a compound [84]. In tests in vitro, this compound antagonizes TRPV1 and functions as a µ-opioid agonist. Similarly, in vivo, it displays a potent antinociceptive effect during the first and second phases of the formalin test in a dose-dependent manner [94]. Moreover, biased agonists have been developed rapidly. Biased agonists preferentially activate G protein-mediated signaling or recruit β-arrestins [95], such as the biased agonist herkinorin [96] and oliceridine (TRV130) [97], which activate the G protein with negligible recruitment of β-arrestin 2 and show less tolerance than morphine [96,97]. However, a few studies showed that fewer side effects induced by biased agonists may be due to the low intrinsic efficacy of G protein activation, but not because of the reduction in recruitment of β-arrestin2 [98]. Therefore, more studies should be conducted to validate the efficiency of biased agonists.

Mechanism-based therapies have also resulted in significant advances. Intracellular scaffold protein β-arrestin 2 is involved in opioid tolerance and opioid-induced hyperalgesia, and bidirectionally regulates TRPV1 and μ-opioid receptors. The antinociception of intrathecal morphine is enhanced by the intrathecal infusion of siRNA against β-arrestin 2, and the tolerance is attenuated simultaneously in rats [99]. Consistent results have also been obtained in β-arrestin 2-deficient mice, which showed an enhanced analgesic effect of morphine, and failed to develop antinociceptive tolerance [11,12]. However, β-arrestin 2 plays a scaffolding role and is not a signaling molecule, and targeting the upstream or downstream molecules of β-arrestin 2 may be efficient to regulate nociception [1]. For example, the downstream target Src family of non-receptor tyrosine kinases is involved in opioid receptor signaling, phosphorylation, endocytosis, and tolerance via the formation of complexes with β-arrestin 2 [100,101]. Src inhibitors can mitigate hyperalgesia, reverse tolerance, and restore analgesia [94,102,103], making them promising candidates for opioid adjuncts.

## 6. Conclusions

In summary, in this paper we discuss the role of TRPV1 in antinociception and its potential to reduce the side effects of opioids, such as opioid tolerance, opioid-induced hyperalgesia, addiction, and inefficiency in the mitigation of neuropathic pain. Indeed, the role of TRPV1 in other opioid side effects, such as respiratory depression, nausea and vomiting, constipation, and some endocrine responses, were not considered in this review because of the shortage of relevant studies. Collectively, regulation of TRPV1 may potentially ameliorate the side effects of opioids and enhance their analgesic efficacy in neuropathic pain.

## Figures and Tables

**Table 1:** The possible mechanisms and therapeutic strategies to alleviate opioid side effects via regulating TRPV1.

Opioid Limitations in Analgesia	Possible Mechanisms	Strategies
Opioid tolerance and Opioid-induced hyperalgesia	μ-opioid desensitization and TRPV1 sensitization; β-arrestin2 bidirectionally regulates μ-opioid and TRPV1 receptors.	Combination drug therapy; Developing dual-acting drugs targeting μ-opioid and TRPV1 receptors.
Addiction	TRPV1 receptors expressed in the dorsal striatum and nucleus accumbens mediate opioid reward.	Blocking TRPV1 receptors expressed in the dorsal striatum and nucleus accumbens is efficient.
Less efficiency to neuropathic pain	Lack of functional μ-opioid receptors following nerve injury and TRPV1 receptor sensitization.	Co-administration of TRPV1 antagonists and opioids in the primary action sites of the periphery.
